# To become part of the team—patient experiences of participating in decision-making for a new treatment (proton beam therapy)

**DOI:** 10.1007/s00520-024-08631-y

**Published:** 2024-06-18

**Authors:** K. Sjövall, K. Ahlberg, P. Fessé, P. Fransson, I. Kristensen, E. Ohlsson-Nevo, L. Åkeflo, U. Langegård

**Affiliations:** 1 Faculty of Health Sciences, Kristianstad University, 291 88 Kristianstad, Sweden; 2 Institute of Health and Care Sciences, Sahlgrenska Academy, University of Gothenburg, Gothenburg, Sweden; 3 Centre for Research and Development, Uppsala University/Region Gävleborg, Gävle, Sweden; 4 Department of Nursing, Umeå University, Umeå, Sweden; 5 Radiation Physics, Department of Haematology, Oncology and Radiation Physics, Lund University Hospital, Lund, Sweden; 6 Department of Oncology, Faculty of Medicine and Health, Örebro University, Örebro, Sweden; 7 Department of Oncology, Sahlgrenska University Hospital, Gothenburg, Sweden

**Keywords:** Proton beam therapy, Radiotherapy, Participation in care, Shared decision making

## Abstract

**Purpose:**

The aim of this study was to explore patients’ experience of participation in the treatment decision of proton beam therapy versus conventional radiotherapy.

**Background:**

Proton beam therapy (PBT) has become a treatment option for some cancer patients receiving radiotherapy. The decision to give PBT instead of conventional radiotherapy (CRT) needs to be carefully planned together with the patient to ensure that the degree of participation is based on individuals’ preferences. There is a knowledge gap of successful approaches to support patients’ participation in the decision-making process, which is particularly important when it comes to the situation of having to choose between two treatment options such as PBT and CRT, with similar expected outcomes.

**Method:**

We conducted a secondary analysis of qualitative data collected from interviews with patients who received PBT for their brain tumor. Transcribed verbatims from interviews with 22 patients were analyzed regarding experiences of participation in the decision-making process leading to PBT.

**Findings:**

Participants experienced their participation in the decision-making process to a varying degree, and with individual preferences. Four themes emerged from data: to be a voice that matters, to get control over what will happen, being in the hand of doctors’ choice, and feeling selected for treatment.

**Conclusion:**

A decision for treatment with PBT can be experienced as a privilege but can also cause stress as it might entail practical issues affecting everyday life in a considerable way. For the patient to have confidence in the decision-making process, patients’ preferences, expectations, and experiences must be included by the healthcare team. Including the patient in the healthcare team as an equal partner by confirming the person enables and facilitates for patients’ voice to be heard and reckoned with. Person-centered care building on a partnership between patients and healthcare professionals should provide the right basis for the decision-making process.

## Background

Proton beam therapy (PBT) has today become a treatment option for cancer patients receiving radiotherapy. For some patients, it means that there are two equivalent treatment options in terms of expected outcomes. As facilities for PBT are less accessible than facilities for conventional radiotherapy (CRT), the option of PBT might entail practical consequences such as living far from home during the treatment period. However, more research is still needed to study the potential benefits of PBT over CRT. Thus, the decision to give PBT instead of CRT needs to be carefully planned together with the patient to ensure that the degree of participation is based on individuals’ preferences.

World Health Organization [1] state that it is the fundamental rights for patients to be involved in their healthcare and treatment in aligning with their individual preferences. In Sweden, this is regulated in the law, emphasizing the right to receive individually adjusted information and have the possibility to choose treatments. The law also includes the right to seek a second opinion [2–4]. Research indicates that patients who perceive themselves as active participants in the decision-making process regarding their treatment are likely to experience less decisional conflict over the treatment decision with improved wellbeing [5, 6]. In cancer care, a higher level of perceived shared decision-making is positively associated with a higher quality of life (QoL) [7], whereas a lower degree of perceived participation has been associated with lower ratings of care quality [8]. However, preferences for participation in treatment decision-making vary among patients [8–10]. As for patients’ definition of participation, participation is related to having knowledge rather than being informed and sharing experiences, as well as sharing decision-making [11]. As for healthcare professionals, patient participation is often related to the process of decision-making [12].

In the European Code of Cancer Practice, shared decision-making (SDM) is stated as one of 10 overarching rights for patients [6]. When having more than one treatment option with equal outcomes, SDM is of high importance. SDM aims to increase and support patient participation in the decision-making process about treatment. It is suggested that SDM is an optimal approach not only when making healthcare decisions, based on both ethical and legal reasons, but also when it comes to improving patient outcomes [5, 6, 13, 14]. SDM is proposed to be a central part of the paradigm shift towards a patient-centered care in the twenty-first century [10, 15]. A frequently employed model for SDM, outlined by Charles et al. [16], describes SDM as the participation of at least two individuals/parties who both need to take steps to participate, meaning that both parties agree that SDM is necessary and preferred. Mutual information sharing is a prerequisite, and the ultimate decision is made and agreed upon by both parties. Thus, the physician and the patient make the decision together based on the best available evidence alongside the patients’ values and preferences [16]. It requires an approach of “patient as a partner” in relation to the healthcare team rather than the paternalistic traditional approach [17–20]. The partnership is shaped by the communicative and relational aspects of the patient-provider relationship [21].

As PBT is often centralized in regional or often national treatment facilities, travel and accommodation are requirements for most patients. This means that treatment involves several weeks from home, with extensive practical consequences in daily life. The decision-making process is often complex, with multiple steps and sometimes several medical contacts. Patients are the experts of their own illness experience [17]. There is a need for increasing our understanding of patients’ perspective of the partnership role [18]. More knowledge is needed to develop approaches to support patients’ participation in the decision-making process when it comes to the situation of having to choose between two treatment options such as PBT and CRT, with similar expected outcomes. In this study, we explore patients’ experiences of participation in the treatment decision of proton beam therapy versus conventional radiotherapy.

## Methods

### Study design

We conducted a secondary analysis of qualitative data collected from interviews with patients who received PBT for their brain tumor. Previously collected data from a primary study was re-used to make a supplementary analysis [22]. The primary study had the aim of exploring the process of symptom management in patients with brain tumor treated with PBT [23]. Both studies are part of the ProtonCare project, developed by the ProtonCare study group (PCSG). PCSG is a national research group with the task of conducting health and care science research in relation to PBT. The overall purpose of the ProtonCare project is to evaluate PBT and PBT-related care from the patients’ perspective by assessing patient-reported outcomes and experiences in patients undergoing PBT or CRT [24].

The primary study was conducted at the Skandion Clinic in Uppsala, Sweden, the pioneering clinic in the Nordic region that provides PBT. Patients are referred to Skandion Clinic for PBT from all over Sweden. A nearby hotel is available for patients who live too far away to commute daily for the treatment.

## Data collection and procedure

Primary data was collected from December 2015 to August 2016. Invitation to the study was made via telephone by the last author (UL). Interviews were conducted by the last author (UL) (28 interviews) or another experienced oncology nurse (6 interviews). Having two experienced interviewers enabled the inclusion of participants from all parts of Sweden. The same interview guide was used by both interviewers, and the interview technique was discussed to ensure dependability. Interviews were conducted at the Skandion clinic or at two of the university hospitals and lasted between 30 and 70 min. The interviews were recorded and then transcribed verbatim by the last author (UL).

The interview started with an open question “Can you please tell me about your situation based on your current illness and the treatment of it?” To invite participants to talk about their experiences regarding participating in decision-making, questions such as “Have you been involved in the decision about the treatment?” and “In what way have you been involved in the decision about your treatment?” were asked. Follow-up questions were asked about the experience of participating or of not participating in the treatment decision process. Clarifying questions were also asked, for example, “Can you tell me more about it?”.

Data collection and recruitment of new participants were closed when saturation was reached. That was the timepoint when the most recent interviews did not seem to make a substantial contribution to what was found in earlier interviews.

## Participants

This study included 22 adult patients with primary brain tumor who were undergoing PBT. Ten participants were interviewed during their treatment, and 12 participants were interviewed immediately after their treatment period. Respondents were strategically selected to ensure consideration for factors such as age, sex, and civil status (as shown in Table 1). All respondents were referred to the Skandion clinic from two university hospitals in Sweden. One patient chose not to participate, explaining that she had time constraints. All patients who accepted participation gave written informed consent.

**Table 1:** Respondents demographic information

	*n* = 22
Gender, *n*	
*Female*	10
*Male*	12
Age, years	
*Mean (range)*	47 (26–74)
Civil status, *n*	
*Single*	6
*Married*	5
*Married with children living at home*	11
Education, *n*	
*Elementary school*	3
*Secondary*	11
*University*	8

### Analysis

Analysis of data was conducted using qualitative content analysis [25]. Transcripts of the interviews were read in their entirety to get a picture of the whole. Text about respondents’ experiences of participating in treatment decision was subsequently extracted and compiled into a single text. It roughly corresponded to one-tenth of the entire transcribed interviews, which constituted the unit of analysis.

In the first step, the text was sorted into content areas defining a rough structure of content. Three content areas were recognized: descriptions of preferences for participation, descriptions of possibilities for participation, and descriptions of limitations for participation. In the second step, the text within each content area was sorted into meaning units. Third, the meaning units were condensed and labelled with a code. Two authors (KS and UL) read and discussed the codes. In the fourth step, the codes were compared for differences and similarities and sorted into tentative categories, which constituted the manifest content. Fourth, the first and last authors discussed the underlying meaning of the categories in relation to patients’ experiences of participation in the decision-making process. The underlying meaning of the categories was formulated into themes. Subsequently, the themes were discussed and negotiated with all the authors. Four themes were agreed upon. Through the whole analysis process, reading was shifted between the whole and the parts of the text. A systematic and reflexive analysis was sought to ensure trustworthiness. All authors ensured the credibility by contributing with different perspectives in an open dialogue throughout the whole process. The themes are described and illustrated with quotes to validate the interpretation.

### Ethical considerations

All procedures performed in studies involving human participants were in accordance with the ethical standards of the institutional and/or national research committee and with the 1964 Helsinki declaration and its later amendments or comparable ethical standards. The primary study was approved by the Regional Ethical Review Board Gothenburg, Sweden (dnr 433–15). All respondents received oral and written information, and written informed consent was collected from all who agreed to participate. The possibility to facilitate psychosocial support was arranged in case talking about experiences related to the cancer diagnosis and the treatment decision released anxiety or revealed psychosocial needs.

## Findings

**Fig. 1:**
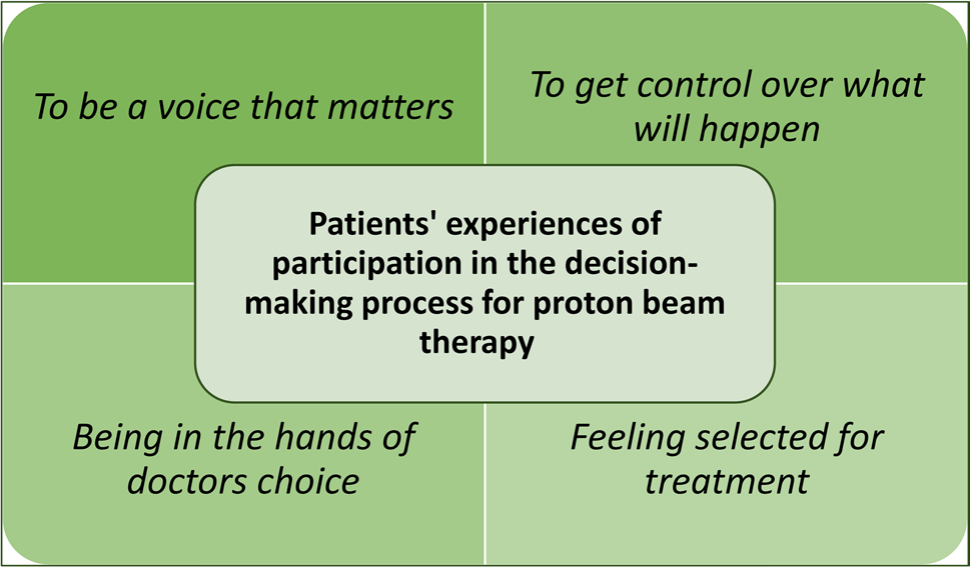
The findings demonstrate participants’ various experiences of participation in the treatment decision-making process for PBT

The findings demonstrate respondents’ various experiences of participation in the decision-making process for PBT. To what extent participation was perceived varied according to individual preferences. Regardless of wanting an active or passive role in the decision-making, participants are consistently expected to receive comprehensive information and get involved in the decision-making process. The findings are presented as four themes, presented in Fig. 1 and the text below.

### To be a voice that matters

The experience of being a voice that matters was linked to being confirmed as a person when encountering the doctor and the healthcare team, regardless of participants’ preferences for the degree of participation in the decision. It was expressed as being an equal partner in the team that was listened to for one’s opinion and preferences.

              *I want to feel that they see and hear me… you can have a discussion with me* (IP19)

Having a feeling of being a part of the decision was also related to having an opportunity to choose another treatment option or to withhold treatment.

Being confirmed as a person was also illustrated by having the opportunity to get a second opinion, and that getting a second opinion was supported by the doctor. No matter what treatment was preferred or decided, the feeling of being acknowledged and supported for one’s preferences was important.

The feeling of being an equal partner felt secure/safe. It was a good feeling to be a part of the healthcare team, and not to be alone with the decision.

*…it feels pretty good, if this goes to hell I haven’t made the decision myself* (IP15)

*so for 99% it was me who took the decision, and it felt good to say ‘this is how we do it’* (IP22)

Some respondents felt excluded from the healthcare team that were to decide about the treatment, being left with unanswered questions or just a brochure to read at home. Living with many unanswered questions about the treatment led to feelings of being a person of no importance. Not being confirmed or respected as a person felt like not having a voice that mattered in the team, despite being the one that was most affected by the treatment decision.

*after all, it is my health* (IP18)

### To get control over what will happen

Decision-making for the treatment was experienced as a process to get the whole picture and to get a sense control of what was going to happen. The opportunity to actively engage in the decision-making was related to having knowledge, which in turn was depending on the given information regarding treatment options, treatment outcomes, side effects, and about practical issues. One way to gain control was to seek information and knowledge about PBT thereby enabling active involvement in the decision-making process regarding the choice of treatment.

*I want to know how things are connected. I don’t accept when you see a doctor who tells you just to trust them *(IP16)

Practical issues related to receiving PBT was of great importance as it affected daily life in a considerable way. Practical issues were about living away from home, travelling, being away from and still taking care of family, taking care of pets and for many participants financial issues.

*The hardest part is probably being so far from home, even if you come home on weekends. It’s still far…and the fact that I have a daughter…the fact that my little daughter doesn’t know really where I am*. (IP17)

Knowing how practical issues would be solved provided a sense of control and sense of security.

*it’s small things, like I have a tumor in my brain, but those practical things, it’s something you can control* (IP14)

### Being in the hands of doctors’ choice

The subtheme *being in the hands of doctors’ choice* was permeated by the feeling of not having the knowledge to be a competent decision-maker and therefore handing over the decision to the doctors, with or without trust. For some participants, it was experienced as having confidence in healthcare and confidence in the decision process. It was expressed as handing over the responsibility for the treatment decision with trust.

*I said I trusted what they think is best for… me because I have no idea* (IP22)

For other participants, it was experienced as being just a receiver of the decision. The information about the two treatment options was presented, but it felt like not being in a position where it was possible to choose.

*if you are an experienced oncologist… the you might think differently, but it is the first time I have a brain tumor* (IP5)

Being in a passive position in the decision-making process was associated with lacking sufficient knowledge of the opportunity to actively participate and an insufficiency of the energy required to obtain further information. It meant having no actual possibility to choose, as the PBT was interpreted to be the very best choice. It would have meant rejecting what the doctor says is the best treatment for you, and thereby the chance to be cured.

*I guess I agree to it… I mean, what is the alternative?… I will do what is suggested* (IP13)

The feeling of having no trust in the decision was related to a lack of understanding of the reasons for the decision. Insufficient information about PBT, coupled with the difficulty in accessing relevant information, contributed to a feeling of being excluded.

### Feeling selected for treatment

Many participants described a feeling of gratitude for being specially selected for having PBT as it was a novel treatment method not available for all patients. It felt like being privileged and like someone that was worth treating, someone worth investing in.

*I understand that I am one of the chosen in some way* (IP12)

*I feel privileged to be allowed to come here (Skandion clinic), I mean I am not a youth either *(IP17)

For some participants, a hope to get PBT was raised when the doctor first presented the option just because it was a new treatment method. The hope was also brought with a concern that it would be CRT instead of PBT. Getting the best treatment (PBT) became a mission for some, a mission worth fighting for. When the decision about PBT was finally reached, it brought huge relief for both the participant and relatives.

## Discussion

In this qualitative study, conducted by secondary analysis of an interview data collection, patients’ experiences of participation in the decision-making of PBT versus CRT were explored. Patients’ experiences related to being confirmed as a person and the sense of having control over what will happen. Feelings of being specially selected for a new treatment were positive, whereas the experience of not being a competent decision-maker and just being a receiver of the decision led to feelings of not having a choice. It was clear that practical issues were of great importance in relation to the treatment decision, based on the premises that PBT is a centralized treatment option with many practical consequences for daily life. It is consistent with findings from a previous study where patients prior to receiving PBT expressed living with many worries—worries about living in an altered context away from home and family, worries about financial consequences and about how they would manage and get support [26]. Practical issues can be of great importance for the patient having PBT, as they relate to the feeling of having control in an otherwise stressful situation. Uncertainties about practical and financial aid during PBT might increase the stress as well as the feeling of being shut out from the decision-making process. Including practical issues early in the process of treatment decision is of utmost importance and must not be overlooked by the healthcare team.

The experience of being in the hand of doctors’ choice was related to being inferior in terms of knowledge. Not having sufficient knowledge is one of the most referred obstacles for participation in decision about treatment [27–30]. However, the individual need for knowledge varied in our study, consistent with previous research [19, 31]. More importantly, it is argued that the possibility to participate in treatment decision is not simply the matter of increasing patients’ knowledge about the disease and treatment, but rather understanding patients’ experiences of his/her illness [17]. It also requires healthcares’ view of patients’ knowledge as equal and complementary [12]. Recognizing and supporting patients’ capabilities, beliefs, and preferences by establishing a trusting relationship may be the most important factor to increase patient participation during the decision-making process, regardless of who controls the decision [31, 32]. Prior studies have shown the patients’ desire to take an active part in the decision-making process, or at least understanding decisions made by others with a trust in the decision-maker [31, 33]. On the other hand, trust in the oncological team might result in a lessened need to be actively participating in the care and instead having a need to hand over the responsibility [33]. The lack of trust in healthcare decision-making process became evident in our study when respondents felt like just being the receiver of the treatment decision. Consequently, the work of building a partnership with the patient and thereby enabling patients’ preference-based participation is crucial. Building the partnership should be based on sharing information, where patients’ narrative of the illness is integrated with healthcare’s biomedical assessment of the disease [20]. Tailoring the care in a way that directly addresses the patients’ needs and preferences requires the center to be on the person rather than the medical encounter [29]. Preference-based patient participation needs to include the match between patients’ preferences and patients’ experiences of mutual communication [9]. The meaning of individual recognition, together with mutual trust and open-mindedness in the partnership, confirms findings within cancer care [19, 28, 31, 33]. Hence, building a trustful relationship based on openness and responsiveness towards the patient seems to be a key factor for the mutual information sharing that is said to be the prerequisite for patient participation and SDM. To build the partnership between patient and healthcare professionals, actively listening to patient’s narrative is crucial.

### Study limitations

Doing a secondary analysis of already collected data means that the possibilities for follow-up in-depth questions are limited. Follow-up questions were used during the interviews to get as rich answers as possible. During the analysis phase, it was not possible to do any adjustments initiated by the result of the analysis, which may have restricted the result somewhat. Another limitation with doing a secondary analysis is that data was collected in 2015/2016. However, no previous study about patients’ experiences of participating in the decision-making process of PBT has been published, and the context for the decision process has not changed in any major aspect. We therefore believe that the findings from this study may be a contribution in increasing the knowledge of the area.

The findings in this study are related to all the decisions that are made daily regarding treatment options and especially the organization of PBT. Accessibility and organization of PBT varies on an international level, which may influence the process of treatment decision and patients’ experiences related to it.

This study focused only on adult patients. Many children are treated with PBT to reduce the risk of late-effect toxicities, and because of that parents might assess and experience practical concerns somewhat differently. It might be the same for adult patients that prognosis and the potential avoidance of late-effect toxicity plays a role in the experience of practical concerns related to PBT. Future studies need to address these questions.

Another limitation of the study is the focus on participation in the treatment decision process solely and not including experiences of participation in the care. Since it was not a focus in the primary study, questions were not raised during the interviews. Future studies need to focus on the broader perspective of patient participation.

Further knowledge is also warranted about how patients’ experiences related to the treatment decision influence the longer term on self-care, follow-up care, and the need for support. The overall purpose of the ProtonCare project is to evaluate PBT and PBT-related care from the patients’ perspective by assessing patient-reported outcomes and experiences in patients undergoing PBT or CRT [24]. Within the project, an ongoing study explores patients’ experiences 5 years after completion of the PBT.

## Conclusion

A decision for treatment with PBT can be experienced as a privilege but can also cause stress as it might entail practical issues affecting everyday life in a considerable way (financial and other practicalities related to staying away from home for several weeks). To have confidence in the decision and the decision-making process, patients’ preferences, expectations, and experiences must be included by the healthcare team. It requires that the patient has access to relevant information and an understanding according to individual preferences. Including the patient in the healthcare team as an equal partner by confirming the person enables and facilitates for patients’ voice to be heard and reckoned with. Person-centered care, a partnership between patients and healthcare professionals, should provide the right basis for the decision-making process.

## Data Availability

No datasets were generated or analyzed during the current study.
